# Dysregulated Gene Expression of Imprinted and X-Linked Genes: A Link to Poor Development of Bovine Haploid Androgenetic Embryos

**DOI:** 10.3389/fcell.2021.640712

**Published:** 2021-03-18

**Authors:** Luis Aguila, Joao Suzuki, Amanda B. T. Hill, Mónica García, Karine de Mattos, Jacinthe Therrien, Lawrence C. Smith

**Affiliations:** Département de Biomédecine Vétérinaire, Centre de Recherche en Reproduction Et Fertilité, Université de Montreal, Saint-Hyacinthe, QC, Canada

**Keywords:** haploidy, androgenetic, embryo, XIST, X-chromosome, KCNQ1 locus, genomic imprinting, bovine

## Abstract

Mammalian uniparental embryos are efficient models for genome imprinting research and allow studies on the contribution of the paternal and maternal genomes to early embryonic development. In this study, we analyzed different methods for production of bovine haploid androgenetic embryos (hAE) to elucidate the causes behind their poor developmental potential. Results indicate that hAE can be efficiently generated by using intracytoplasmic sperm injection and oocyte enucleation at telophase II. Although androgenetic haploidy does not disturb early development up to around the 8-cell stage, androgenetic development is disturbed after the time of zygote genome activation and hAE that reach the morula stage are less capable to reach the blastocyst stage of development. Karyotypic comparisons to parthenogenetic- and ICSI-derived embryos excluded chromosomal segregation errors as causes of the developmental constraints of hAE. However, analysis of gene expression indicated abnormal levels of transcripts for key long non-coding RNAs involved in X chromosome inactivation and genomic imprinting of the KCNQ1 locus, suggesting an association with X chromosome and some imprinted loci. Moreover, transcript levels of methyltransferase 3B were significantly downregulated, suggesting potential anomalies in hAE establishing *de novo* methylation. Finally, the methylation status of imprinted control regions for XIST and KCNQ1OT1 genes remained hypomethylated in hAE at the morula and blastocyst stages, confirming their origin from spermatozoa. Thus, our results exclude micromanipulation and chromosomal abnormalities as major factors disturbing the normal development of bovine haploid androgenotes. In addition, although the cause of the arrest remains unclear, we have shown that the inefficient development of haploid androgenetic bovine embryos to develop to the blastocyst stage is associated with abnormal expression of key factors involved in X chromosome activity and genomic imprinting.

## Introduction

In contrast to lower animal classes that can develop from a single parent by parthenogenesis, mammals have developed parental-specific epigenetic strategies such as genomic imprinting that require the contribution from the paternal and maternal genomes for the embryo to develop fully to term. For instance, during preimplantation development the maternal and paternal epigenetic information, represented mainly by DNA methylation and posttranslational histone modifications, are extensively reprogrammed. During post-fertilization reprogramming, the embryo loses gamete-specific DNA methylation patterns inherited from the oocyte and the sperm as it progresses toward the blastocyst stage and gain pluripotency, which occurs in two phases. At the one-cell stage (zygotes), the paternal genome is initially actively demethylated (Gu et al., 2011; Wossidlo et al., 2011); the two parental genomes then undergo gradual genome-wide loss of DNA methylation (Howell et al., 2001), except for imprinted genes and certain repeats, and then gets remethylated after implantation (Messerschmidt et al., 2014), reaching a low point at the blastocyst stage, which is followed by DNA (cytosine-5)-methyltransferase 3A (DNMT3A)-mediated and DNMT3B-mediated *de novo* DNA methylation after blastocyst implantation (Chen and Zhang, 2020).

Nonetheless, despite that normal embryonic development requires contributions from both the maternally and paternally inherited haploid genomes, early development can be achieved from uniparental embryos in mammals using artificial oocyte activation and/or micromanipulation techniques, and these have been extremely useful in delineating genomic function, imprinting status and parental-specific roles in ontogenesis (Cruz et al., 2008; Hu et al., 2015b).

Diploid androgenetic and gynogenetic/parthenogenetic embryos possess two sets of paternal or maternal genomes, respectively, while their haploid counterparts contain only one paternal or maternal genome. Although haploid development is a normal part of the life cycle for some animals (e.g., parasitic wasps), haploidy in mammals is restricted to gametes, which are structurally specialized for fertilization and mitotically incompetent (Shuai and Zhou, 2014).

Uniparental haploid embryos are efficient models for genome imprinting research and allow studies on the contribution of the paternal and maternal genome to early embryonic development. Moreover, haploid embryos have been used to derive embryonic stem cells and hold great promise for functional genetic studies and animal biotechnology (Panneerdoss et al., 2012; Kokubu and Takeda, 2014; Bai et al., 2016, 2019).

Although haploid embryonic stem cells (hESCs) have been obtained in several mammals (Leeb and Wutz, 2011; Yang et al., 2013; Zhong et al., 2016), most reports have indicated poor rates of blastocyst formation and thus flawed inner cell mass formation, indicating constraints at early stages of embryonic development. In mice, studies have revealed that the preimplantation developmental potential of haploids is significantly impaired relative to diploid embryos, due mainly to the disruption of gene regulatory mechanisms (Latham et al., 2002) and abnormal imprinted gene expression (Hu et al., 2015b). However, there are very few studies characterizing the causes of limited development of haploid androgenetic embryos (hAE) in other mammalian models, particularly in domestic species, where the androgenetic embryonic stem cells would provide an useful route for genetic improvement and manipulation (Lagutina et al., 2004; Matsukawa et al., 2007; Park et al., 2009; Vichera et al., 2011).

The generation of mammalian hAE has been achieved by using a variety of methods. In mice, haploidy has been successfully obtained by the bisection of zygotes after fertilization (Tarkowski and Rossant, 1976), the removal of the maternal nucleus from fertilized eggs at the pronuclear stage (Yang et al., 2012), and the injection of sperm into enucleated oocytes (Li et al., 2012; Yang et al., 2012). However, efforts to visualize and enucleate zygotes at pronuclear stages in domestic species is hampered by the presence of dense lipid vesicles, and, thus, removal of the oocyte metaphase spindle is usually performed before *in vitro* fertilization (IVF), as in the case of SCNT, at approximately 16–17 h after beginning of *in vitro* maturation (MII enucleation) (Ross and Cibelli, 2010). Otherwise, enucleation can be performed at the telophase to anaphase transition of meiosis II using the second polar body protrusion as a reliable indicator of the position of the oocyte spindle and its effective microsurgical removal (Bordignon and Smith, 1998; Kuznyetsov et al., 2007; Sagi et al., 2019).

Therefore, our aims were to establish an efficient method to produce bovine hAE and identify the potential causes of their severely limited developmental potential. Our results indicate that the developmental restriction of bovine androgenetic haploid embryos occurs at the time of major transcriptional activation at the 8-cell stage, and during compaction at the morula stage, both of which are associated with the altered expression of key epigenetically regulated genes. The significance and possible explanations for these findings are discussed.

## Materials and Methods

### Oocyte Collection and *in vitro* Maturation

Bovine ovaries were obtained from a local slaughterhouse and transported to the laboratory in sterile 0.9% NaCl at 25–30°C in a thermos bottle. Cumulus–oocyte complexes (COCs) were aspirated from 5 to 10 mm antral follicles using a 12-gauge disposable needle. For *in vitro* maturation (IVM), COCs with several cumulus cell layers were selected, washed and placed in maturation medium composed of TCM199 (Invitrogen Life Technologies), 10% fetal bovine serum (FBS; Invitrogen Life Technologies), 0.2 mM pyruvate (Sigma-Aldrich), 50 mg/mL gentamicin (Sigma-Aldrich), 6 μg/mL luteinizing hormone (Sioux Biochemical), 6 μg/mL follicle-stimulating hormone (Bioniche Life Science) and 1 μg/mL estradiol (Sigma-Aldrich). *In vitro* oocyte maturation was performed for 22–24 h at 38.5°C in a humidified atmosphere at 5% CO_2_.

### Sperm Preparation

Straws of non-sexed and sex-sorted semen stored in liquid nitrogen were thawed for 1 min in a water bath at 35.8°C, added to a discontinuous silane-coated silica gradient (45 over 90% BoviPure, Nidacon Laboratories AB), and centrifuged at 600 × *g* for 5 min. The supernatant containing the cryoprotectant and dead spermatozoa were discarded, and the pellet with viable spermatozoa was re-suspended in 1 mL of modified Tyrode’s lactate (TL) medium and centrifuged at 300 × *g* for 2 min.

### *In vitro* Fertilization

After 20–24 h of IVM, COCs were washed twice in TL medium before being transferred in groups of 5–48 μl droplets under mineral oil. The IVF droplets consisted of modified TL medium supplemented with fatty-acid-free BSA (0.6% w/v), pyruvic acid (0.2 mM), heparin (2 μg/mL) and gentamycin (50 mg/mL). COCs were transferred to IVF droplets 15 min prior to adding the spermatozoa. To stimulate sperm motility, penicillamine (2 mM; Sigma-Aldrich), hypotaurine (1 mM; Sigma-Aldrich) and epinephrine (250 mM; Sigma-Aldrich) were added to each droplet. The selected spermatozoa were counted using a hemocytometer and diluted with IVF medium to obtain a final concentration of 1 × 10^6^ sperm/mL. Finally, 2 μL of the sperm suspension was added to the droplets containing the matured COCs. The fertilization medium was incubated at 38.5°C for 18 h in a humidified atmosphere of 95% air and 5% CO_2_. Presumptive zygotes were denuded by treatment with 0.1% bovine testicular hyaluronidase.

### Intracytoplasmic Sperm Injection

Intracytoplasmic sperm injections (ICSI) were performed according to standard protocols (Horiuchi et al., 2002) on the stage of a Nikon Ti-S inverted microscope (Nikon Canada Inc.) fitted with Narishige micromanipulators (Narishige International) and a Piezo drill system (PMM 150HJ/FU; Prime Tech Ltd.). Before ICSI, oocytes were denuded of granulosa cells by gently pipetting in the presence of 1 mg/mL hyaluronidase, selected for the presence of the first polar body and randomly allocated to experimental groups. After ICSI, oocytes were washed at least three times and cultured in modified synthetic oviduct fluid (mSOF) media as previously described by Landry et al. (2016).

### Production of Haploid Embryos

Bovine hAE produced by IVF were enucleated by removing mechanically the oocyte chromosomes (enucleation) either before or after insemination. When enucleating before IVF, COCs were denuded at 24 h after IVM, the oocytes were then exposed 10 min to 5 μg/mL cytochalasin B and 10 μg/mL Hoechst 33342 (Thermo Fisher) and a small portion (±10%) of the cytoplasm surrounding the first polar body was removed by aspiration into a micropipette. The aspirated cytoplasmic bleb was observed under UV light to ascertain whether the metaphase II spindle (MII) had been properly removed at enucleation. Oocytes in which enucleation was performed after IVF were removed from the fertilization droplet at different times after insemination, denuded of the cumulus cells by gentle pipetting and those presumptive zygotes with recently extruded second polar bodies were placed in cytochalasin B (Sigma-Aldrich) and Hoechst 33342 for 15 min as described above. A cytoplasm portion (±10%) surrounding the second polar body was aspirated from the oocyte, checked for the presence of a telophase-stage (TII) spindle, washed and returned to *in vitro* culture medium droplets.

In a second approach, hAE produced by ICSI were obtain by removing the oocyte TII spindle after 4 h post-ICSI. Enucleated zygotes were cultured as described above. Parthenogenetic embryos were produced according (Ock et al., 2003). Briefly, chemical oocyte activation was performed between 20 and 24 h after IVM by 5 min exposure to 5 μM ionomicyn (Calbiochem). To obtain haploid parthenotes, ionomycin treatment was followed by incubation in 10 mg/mL cycloheximide (CHX; Sigma-Aldrich) for 5 h, which enables complete extrusion of the second polar body. For diploid parthenogenotes, ionomycin-activated oocytes were exposed for 5 h to CHX and 5 mg/mL of cytochalasin B to inhibits the extrusion of the second polar body and, thereby, induce diploidization. After parthenogenetic activation, haploid or diploid parthenotes were washed and allocated to *in vitro* culture drops.

### *In vitro* Culture

For *in vitro* culture, groups of 10 embryos were placed in droplets (10 μl) of modified synthetic oviduct fluid (mSOF) with non-essential amino acids, 3 mM EDTA, and 0.4% fatty-acid-free BSA (Sigma-Aldrich) under embryo-tested mineral oil. The embryo culture dishes were incubated at 38.5°C with 6.5% CO_2_, 5% O_2_, and 88.5% N_2_ in saturation humidity. Cleavage rate was recorded at 48 h (Day 2) of culture (IVF and ICSI = Day 0). Morula and blastocyst development rate were recorded on days 6 and 7 post-fertilization, respectively. Some haploid embryos were cultured for an extra 24 h to determine blastocyst rates at day 8 (192 h after ICSI). After assessment of development, embryos were either fixed for cell number evaluation or snap-frozen in liquid N_2_ and stored at −80°C for RNA extraction.

### Assessments of Pronuclear Formation and Total Cell Number

Pronuclear formation was assessed at 18–20 h after activation or fertilization and embryo quality was assessed on the basis of morphology and total cell number. Briefly, embryos at day 7 were classified morphologically as morula (compacted and >32 cells), early blastocyst (<200 μm), expanded blastocyst (>200 μm), and hatched blastocyst (after complete extrusion from the zona pellucida). Embryos at different stages were fixed overnight in paraformaldehyde and stained with Hoechst 33342 (10 μg/mL) for 15 min, and total number of cells and pronuclear formation were observed and analyzed by fluorescence microscopy (Axio Imager M1, Zeiss).

### Karyotype Analysis

After culture in the in presence of 0.05 μg/mL of Colcemid (KaryoMax^®^ Life Technologies) for 5 h, morula-stage embryos obtained at Day-6 of culture were exposed to a hypotonic (0.75 M KCl; Sigma-Aldrich) solution for 10 min to induce swelling. Subsequently, embryos were placed on a clean glass slide in a small volume of medium and fixed using an ethanol–acetic acid solution (1:1; v/v) that was dropped on the embryos while gently blowing the slides for 15 min to allow drying under observation on a stereoscope. After drying, slides were stained with Hoechst 33342 (10 μg/mL) for 15 min. Chromosome spreads were evaluated at 1,000× magnification using oil immersion optics and fluorescence microscopy (Axio Imager M1, Zeiss). Embryonic cells were classified as haploid (*n* = 30), diploid (*n* = 60), or aneuploid (*n* ≠ 30 or 60) according to the number of chromosomes.

### RNA Extraction and RT-PCR

For analysis of gene expression, embryos were pooled for each stage of development: 15 for 8-cell stage and 5 for morulae. Each group was carried out in three biological replicates and each replicate was run in duplicate. Total RNA from the pooled embryos was extracted using the Arcturus PicoPure RNA Isolation kit (Life Technologies) and reverse transcribed into cDNA using SuperScript Vilo (Invitrogen). Quantitative RT-PCR was performed using the RotorGene SyBr Green PCR kit (Qiagen) in a Rotor-Gene Q PCR cycler under the following amplification conditions: 95°C for 5 min, followed by 40 cycles at 95°C for 5 s and at 60°C for 10 s. Primers were designed using Oligo6 software and the geometric means of three reference genes (GAPDH, ACTB, and SF3A) were used for normalization. The stability of the reference genes across our samples was confirmed using Bestkeeper (Pfaffl et al., 2004). A list of all primers used can be found in Supplementary File 1.

### Gene Specific Bisulfite Sequencing

Gene specific bisulfite sequencing was performed in morula (pools of two) and blastocyst (individually) stages. For control groups (somatic cells, sperm, biparental, and parthenogenetic embryos), analysis was done in one or two biological replicates. For the haploid androgenetic group, it was carried out in three biological replicates. Genomic DNA extraction and bisulfite treatment were done using a kit (EZDNA methylation-direct kit, Zymo research). Primers specific for bisulfite-converted DNA were designed within the DMR region of XIST (gene ID: 338325), KCNQ1OT1 (gene ID: 112444897), and H19 (gene ID: 100126192). KCNQ1OT1: F: GGTTAGAGGAGTATTTTGAAG AGA, R: TCAACCCTCTCAACCAATAA, XIST: F: TTTTGTTG TAGGGATAATATGGTTGA, R: TCATCTAATTCCATCCTCC ACTAACT, and for H19: F: TATTTTAGATAGGGTTGAGAGG TTG, R: CAAACATAAAAATCCCTCATTA-TCC. Each PCR reaction was performed in triplicate. The PCR reaction was carried out in a final volume of 50 μL containing 1–2 μL of bisulfite-treated DNA, 0.2 μM each primer, 0.3 mM mixed dNTP, 1X PCR buffer, 1.5 mM MgCl_2_ with 2U of Platinum Taq DNA Polymerase (Invitrogen). The reactions were performed using an initial 2-min step at 94°C followed by 45 cycles of 30 s at 94°C, 30 s at 53°C, 1 min at 72°C, and a final 5 min step at 72°C. The PCR products were resolved in 1.2% agarose gels, followed by purification using the QIAquick Gel Extraction kit (Qiagen). Purified fragments were pooled and subcloned in pGEM-T Easy Vector (Promega). At least 20 clones for each sample were picked and sequenced. Validation of the imprinted status of each DMR was performed as previously described by Lafontaine et al. (2020), by assessing methylation of sperm DNA (expected methylation >90% or <10%) and fibroblast cell DNA (40–60% expected methylation).

### Statistical Analysis

Quantitative data sets are presented as means and standard deviation (±S.D) and analyzed using one-way ANOVA. *Post hoc* analysis to identify differences between groups was performed using Tukey test. Binomial data sets, such as pronuclear formation, were analyzed by using Fisher test. Differences were considered significant at *p* < 0.05.

## Results

### Haploid Androgenetic Embryos Produced by Enucleation After Fertilization Leads to Better Development, but It Is Unreliable Due to High Polyspermy Levels

Since the resumption of meiosis in the oocyte by the fertilizing spermatozoa can vary significantly when using conventional IVF, oocytes were exposed to spermatozoa during different time periods to identify an optimal fertilization time at which the second polar body could be used to locate the spindle for enucleation. With this purpose, we evaluated developmental potential after removing presumptive zygotes from the IVF drops at different times after insemination. Results indicated that second polar bodies were present in 80% of the oocytes by 6 h or more post insemination (hpi) while only 50% were fertilized with insemination periods of 4 hpi or less. Moreover, removal the presumptive zygotes from the IVF drop after 6 hpi led to better preimplantation development when compared to the shorter exposure periods to spermatozoa (*p* < 0.05), indicating that a 6 hpi period was suitable for oocyte enucleation post fertilization (data not shown).

Having identified an optimal period to expose oocytes to spermatozoa for enucleations during extrusion of the second polar body, we performed an experiment to compare the developmental outcome of putative haploid zygotes that were denuded and enucleated either pre- ot post-IVF. Diploid controls, i.e., denuded but non-enucleated pre- and post-fertilization groups, were cultured concomitantly. Confirming previous results (Lagutina et al., 2004; Vichera et al., 2011), cleavage rates at 48 h did not differ between putative haploid and control diploid embryos, indicating that the first cell divisions are not affected by haploidy or the timing of enucleation. However, blastocyst development was significantly reduced in haploid embryos, indicating that androgenetic haploidy disturbs early development beyond the first cleavage. Nonetheless, we found that instead of performing enucleation at MII, enucleations after IVF produced significantly more 8-cell (*p* < 0.05) at Day 2 and blastocyst (*p* < 0.01) stage embryos at Day 7 after IVF, indicating that enucleation of the oocyte’s spindle before fertilization is more detrimental to the development of haploid androgenetic embryos (Table 1).

**TABLE 1:** Development to cleavage and blastocyst stages at Day-2 (48 h) and Day-7 (168 h) post insemination (hpi) of control and putative androgenetic embryos manipulated both before (pre-IVF) and after (post-IVF) *in vitro* fertilization (IVF).

Groups	No. oocytes	Embryo development			
		Cleaved	%	Blastocyst day 7	%
Control denudation 6 h after IVF	126	91	72%	35	28%
Haploid enucleated after IVF	186	149	80%	20	11%
Control denudation 2 h before IVF	148	112	76%	45	30%
Haploid enucleated before IVF	240	160	67%	4	2%***

*Control denudation 6 h after IVF, oocytes denuded 6 h after insemination. Haploid enucleated after IVF, oocytes denuded and enucleated at 6 h after insemination. Control denudation 2 h before IVF, oocytes denuded at 2 h before insemination. Haploid enucleated before IVF, oocytes denuded and enucleated at 2 h before insemination. Asterisks denote significant differences within columns (p < 0.05).*

Next, we performed DNA staining of the putative haploid zygotes at 20 h after insemination to examine the number of pronuclei of control and enucleated groups (Table 2). The presence of more than one pronucleus in enucleated groups and more than two pronuclei in control zygotes is indicative of polyspermy and/or mitotic errors, both causes of uncertainty with regard to ploidy in presumptive haploid (parthenogenetic and androgenetic) zygotes. No significant differences were observed in the level of multinucleated zygotes, neither between oocytes enucleated before and after-IVF (17% vs. 34%, *p* = 0.08), nor between enucleated and control groups (*p* = 0.07). Nonetheless, approximately one fifth and one third of the putative haploid zygotes derived from enucleated oocytes before and after-IVF, respectively, contained two or more pronuclei. These results clearly indicate that the production of bovine hAE by conventional IVF protocols leads to significant uncertainty with regard to ploidy. Therefore, a more reliable and less error prone method was required to efficiently eliminate the possibility of polyspermic fertilization when deriving haploid androgenetic zygotes.

**TABLE 2:** Formation of pronuclei of zygotes fixed at 20 h after insemination in control and enucleated oocytes manipulated either before or after insemination.

Group	Oocytes (n)	Pronuclear Formation 20 hpi (no. and %)						
		1 PB			Others			
		1 PN	2 PN	PDSH	CSH	≥3 PN	0 PN	*Multinucleate
Control denuded 6 h after IVF	64 (3)	0 (0%)	44 (69%)*	0 (0%)	0 (0%)	14 (22%)	6 (9%)	14 (22%)
Haploid enucleated after IVF	80 (3)	43 (54%)*	6 (8%)	0 (0%)	0 (0%)	21 (25%)	10 (13%)	27 (34%)
Control denuded 2 h before	10 (2)	0 (0%)	8 (80%)*	0 (0%)	0 (0%)	1 (10%)	1 (10%)	1 (10%)
Haploid enucleated before IVF	12 (2)	9 (75%)*	0 (0%)	0 (0%)	0 (0%)	2 (17%)	1 (8%)	2 (17%)

PB, polar body; PN, pronuclei; PDSH, partially decondensed sperm head; CSH, condensed sperm head. Control denudation 6 h after IVF, oocytes denuded 6 h after insemination. Haploid enucleated after IVF, oocytes denuded and enucleated at 6 h after insemination. Control denudation 2 h before IVF, oocytes denuded at 2 h before insemination. Haploid enucleated before IVF, oocytes denuded and enucleated at 2 h before insemination. *Multinucleated, multinucleated zygote was assumed as the presence of more than one pronucleus in haploid groups and more than 2 pronuclei in biparental zygotes. Asterisks denote significant differences within columns (p < 0.05).

### Haploid Androgenetic Embryos Can Be Obtained Reliably by Intracytoplasmic Sperm Injection Followed by Enucleation of the Telophase II Spindle

**FIGURE 1:**
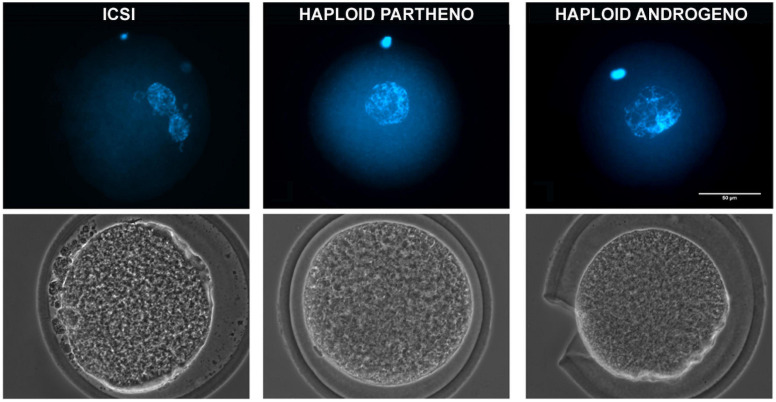
Representative images of a 1-cell stage zygote fixed at 20 h after activation showing DNA staining (upper) and phase-contrast images (lower) of a ICSI biparental embryo (2 pronuclei), a haploid parthenogenetic embryo (1 female pronucleus; obtained by oocyte activation using ionomycin followed by cyclohexymide), and a haploid androgenetic embryo (1 male pronucleus; obtained by ICSI and oocyte enucleation). ICSI, intracytoplasmic sperm injection using female-sorted semen. Scale bar = 50 μm.

Due to the unreliability of conventional IVF in deriving bovine hAE, we next examined the use of intracytoplasmic sperm injection (ICSI) to eliminate the possibility of polyspermic fertilization. Since better development was achieved by enucleation after fertilization when using conventional IVF, we performed ICSI followed by enucleation 3–4 h later, i.e., when the telophase-II spindle and the second polar body were easily identified for microsurgical removal. In order to verify the efficiency of the enucleation procedure after ICSI to produce hAE, we evaluated the rate of pronuclear formation at 20 h post ICSI (Figure 1 and Table 3) and used the parthenogenetic haploid zygotes as a positive control for prescence of only one pronucleus.

**TABLE 3:** Formation of pronuclei and other chromatin structures in diploid (ICSI) and haploid zygotes (parthenogenetic and androgenetic) observed at 20 h after activation.

Group	Oocytes (n)	Pronuclear Formation 20 hpi (no. and %)						
		1 PB			Others			
		1 PN	2 PN	PDSH	CSH	≥3 PN	0 PN	*Multinucleate
ICSI	23 (2)	0 (0%)	18 (78%)*	1 (4%)*	4 (17%)*	0 (0%)*	0 (0%)	0 (0%)
Haploid partheno	35 (3)	30 (86%)*	0 (0%)	0 (0%)	0 (0%)	5 (14%)	0 (0%)	5 (14%)**
Haploid androgeno	92 (7)	59 (64%)*	0 (0%)	12 (13%)**	20 (22%)**	1 (1%)**	0 (0%)	1 (1%)

ICSI, intracytoplasmic sperm injection. Haploid partheno, haploid parthenogenetic embryo obtained by oocyte activation using ionomycin followed by cyclohexymide. Haploid androgeno: haploid androgenetic embryo obtained by ICSI and TII enucleation. PB, polar body; PN, pronuclei; PDSH, partially decondensed sperm head. CSH, condensed sperm head. Asterisks denote significant differences within columns (p < 0.05). *Multinucleated: multinucleated zygote was assumed as the presence of more than one pronucleus in haploid groups and more than 2 pronuclei in biparental zygotes. Asterisks denote significant differences within columns (p < 0.05).

DNA staining indicated a similar percentage of zygotes showing incomplete decondensation of the sperm head at 20 h after ICSI in the enucleated embryos compared to controls (35% vs. 21%, *p* > 0.05), indicating that the removal of the oocyte telophase II spindle at the first hours after ICSI does not disturbs neither sperm head decondensation nor the formation of the paternal pronucleus. Moreover, 99% of the zygotes reconstructed by enucleation after ICSI had only one chromatin structure (Table 3 and Figure 1), confirming that ICSI eliminates the possibility of polyspermic fertilization and is, therefore, more reliable than conventional IVF for deriving bovine haploid androgenetic zygotes.

### Haploid Androgenetic Embryos Develop Poorly and Slowly to the Blastocyst Stage

**FIGURE 2:**
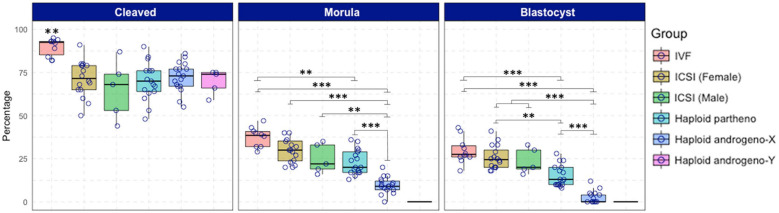
Development to cleavage (Day-2) and blastocyst (Day-7) stages of embryos produced by *in vitro* fertilization (IVF), intracytoplasmic sperm injection (ICSI) using non-sexed and sexed sperm, haploid parthenogenetic and androgenesis using sexed spermatozoa. IVF, *in vitro* fertilization. ICSI (female): intracytoplasmic sperm injection using Y-chromosome sorted sperm. ICSI (male), intracytoplasmic sperm injection using X-chromosome sorted sperm. Haploid partheno, haploid parthenogenetic embryo obtained by oocyte activation using ionomycin followed by cyclohexymide. Haploid androgeno-X, haploid androgenetic embryos generated using X-chromosome sorted sperm. Haploid andro-Y, haploid androgenetic embryos generated using Y-chromosome sorted sperm. n.a. data is not available. Asterisks denote significant differences within columns (^∗^*p* < 0.05, ^∗∗^*p* < 0.01, ^****^p < 0.0001).

**FIGURE 3:**
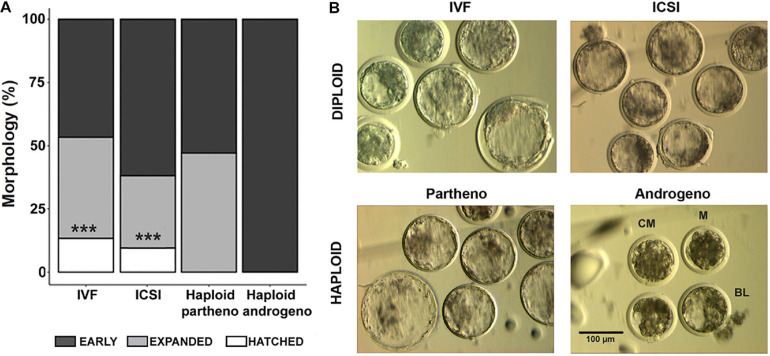
Morphological assessment of haploid and diploid embryos at Day-7 (168 h) of culture. **(A)** Percentage of differing blastocyst stages. **(B)** Representative images of the most advanced embryos from different controls and haploid groups. IVF, *in vitro* fertilized; ICSI, intracytoplasmic sperm injection using female-sorted semen; Haploid partheno, haploid parthenogenetic embryos obtained by oocyte activation using ionomycin followed by cyclohexymide; haploid androgeno, haploid androgenetic embryo obtained by ICSI + oocyte enucleation. M, compact morula; CB, cavitating blastocyst; BL, blastocyst. ^∗∗∗^*p* < 0.001. Scale bar = 100 μm.

**FIGURE 4:**
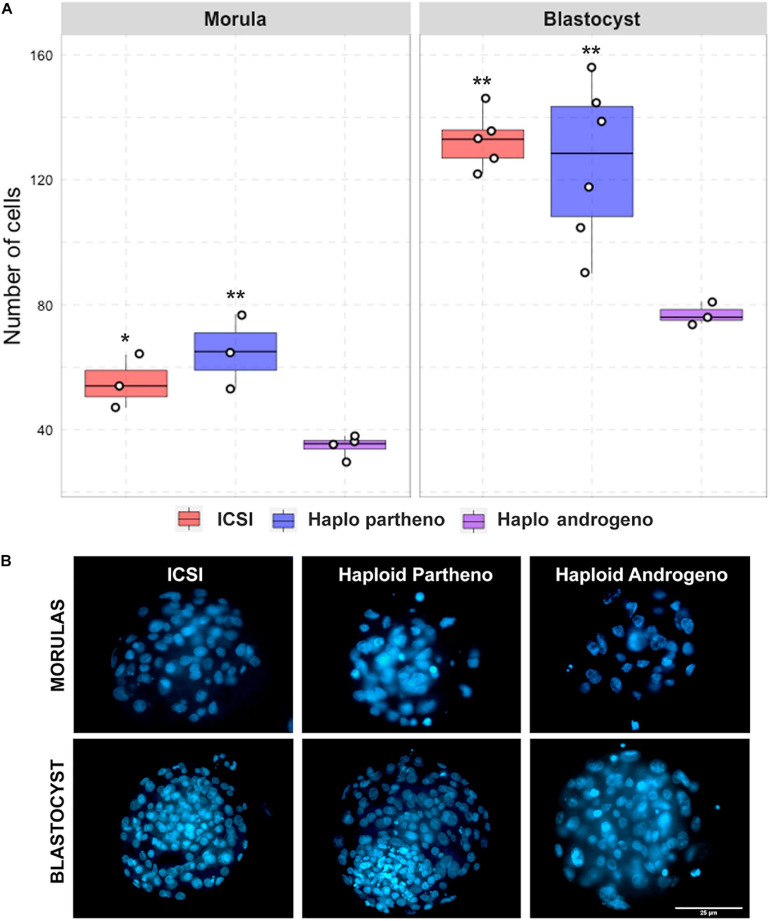
Cell number of diploid and haploid morula and blastocyst stage embryos. **(A)** Nuclear counts and **(B)** representative images of morula and blastocyst stage embryos harvested at Day-6 (144 h) and 168 h (Day-7) of culture, respectively. IVF, *in vitro* fertilized; ICSI, intracytoplasmic sperm injection using female-sorted semen; Haploid partheno, haploid parthenogenetic embryos obtained by oocyte activation using ionomycin followed by cyclohexymide; haploid androgeno, haploid androgenetic embryo obtained by ICSI + oocyte enucleation. ^∗^*p* < 0.05, ^∗∗^*p* < 0.01. Scale bar = 25 μm.

Once the reliability of the ICSI approach for deriving hAE was verified, we next compared early developmental rates of haploid and diploid control groups at different times of *in vitro* culture. Because previous reports have shown that androgenetic embryos produced using Y chromosome-carrying sperm are unable to support development to the blastocyst stage (Latham et al., 2000; Latham et al., 2002; Yang et al., 2012), we used X or Y chromosome-sorted semen to obtain female or male zygotes, respectively. Except for the IVF group that showed the highest cleavage rate (90%; *p* < 0.01), all the remaining groups showed similar levels of cleavage (range 72–74%) (Figure 2 and Supplementary File 2). However, the development of embryos to the 8-cell stage after 48 h of culture (Supplementary Figure 1) was lower only in haploid parthenogenetic embryos, suggesting that manipulation procedures involved in generating hAE do not affect early cleavage events. Although the development to the morula and blastocyst stages was similar between the biparental IVF and ICSI controls, haploid parthenogenetic embryos showed similar development to the morula stage, but fewer percent achieved the blastocyst stage when compared to biparental embryos, indicating a negative effect of maternal haploidy (Figure 2 and Supplementary File 2). On the other hand, hAE showed the lowest developmental potential (9 and 3% for morula and blastocyst stage, respectively; *p* < 0.0001) compared to biparental and the haploid parthenogenetic group (Figure 2 and Supplementary File 2), indicating that androgenetic haploidy is much less suitable for supporting early development when compared to the parthenogenetic haploidy. Besides, only hAE produced with sperm carrying X-chromosome reached the morula and blastocyst stages (Figure 2 and Supplementary File 2). DNA staining showed that hAE produced with sperm carrying the Y-chromosome did not develop beyond 20 cells (Supplementary Figure 2). Further assessment of embryo morphology at Day-7 indicated major differences in the percentual expansion and hatching of haploid androgenetic blastocyst stage embryos, indicating defective formation of the blastocoel cavity (Figure 3). Moreover, assessment of nuclear number of Day-6 morulae and Day-7 blastocysts showed that androgenotes contained significantly fewer cells when compared to ICSI control and haploid parthenotes of the same age (Figure 4), indicating delayed cell division. Actually, some androgenetic embryos only reached the blastocyst at Day-8, indicating that the developmental steps required for blastulation are delayed in the few hAE embryos where development persists to the blastocyst stage (Data not shown). After cleavage, hAE underwent developmental arrest concurrently with zygote genome activation, and only 13% of the cleaved embryos progressed to morula stage, compared to haploid parthenotes (32%) and diploid groups (>40%) (Figure 2 and Supplementary File 2). In addition, haploid androgenotes arrested once again at morula stage when only 26% of the Day-6 morula became a blastocyst (Figure 2 and Supplementary File 2). Since the ratio of haploid parthenogenetic morulae (68%) that become blastocyst was significantly higher (*p* < 0.005) than the haploid androgenetic group, these results indicate that, as in the other mammalian models, the bovine haploid paternal condition is less capable to support early embryonic development compared to its maternal counterpart.

### Haploid Androgenetic Morula-Stage Embryos Maintain Stable Ploidy

**FIGURE 5:**
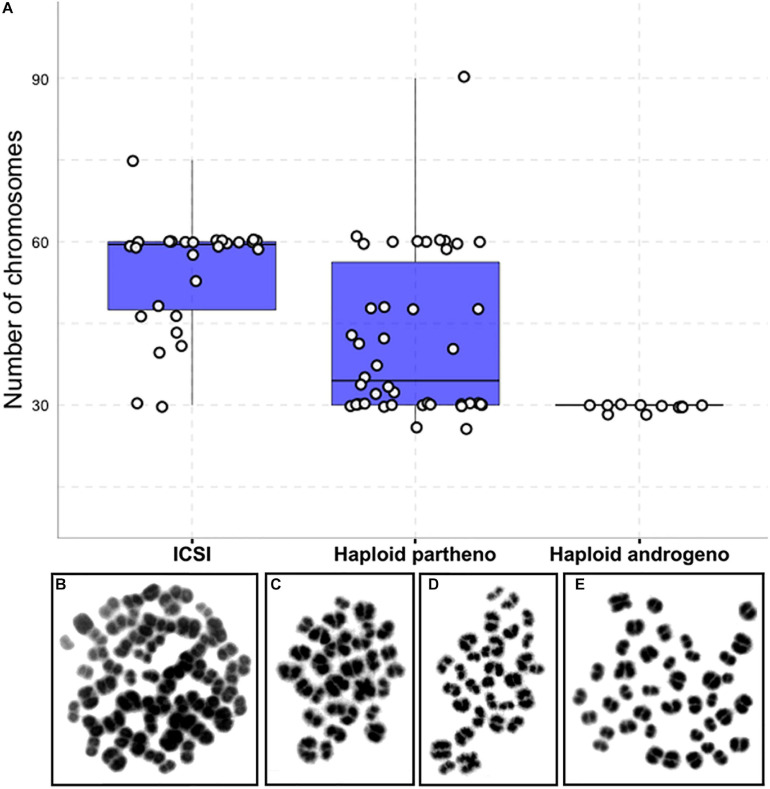
Karyotype analysis of haploid and diploid morula stage (Day-6) embryonic blastomeres. **(A)** Group chromosomal number distributions showing mean values (blue horizontal lines) with standard deviations (red vertical lines). **(B–E)** Representative images of DAPI-stained chromosomal spreads of embryonic blastomeres from **(B)** diploid ICSI (60 chromosomes), **(C)** haploid parthenote (30 chromosomes), **(D)** haploid androgenote (30 chromosomes), and **(E)** aneuploid (40 chromosomes) parthenote embryo. ICSI, intracytoplasmic sperm injection using female-sorted semen; haploid partheno, haploid parthenogenetic embryos obtained by oocyte activation using ionomycin followed by cyclohexymide; haploid androgeno, haploid androgenetic embryo obtained by ICSI + oocyte enucleation.

Previous reports have shown that chromosomal anomalies occur often in embryos manipulated *in vitro* (Kawarsky et al., 1996; Rubio et al., 2003; Ross et al., 2008). Therefore, we decided to verify whether the ploidy of the haploid androgenetic embryos was particularly aberrant via karyotyping of morula-stage embryos obtained at Day-6 of culture. Biparental ICSI and haploid parthenogenetic embryos contained a large percentage (54 and 64%, respectively) of abnormal karyotypes (Table 4 and Figure 5). In contrast, most of the haploid androgenetic blastomeres analyzed (81%) contained a normal haplotype (1*n* = 30), which contrasted, but not significantly (*p* > 0.05), with the biparental ICSI and haploid parthenogenetic embryos, that contained fewer (46 and 35%, respectively) normal karyotypes (Table 4 and Figure 5). These results indicate a stable chromosomal alignment and segregation during early development in hAE which contrasts with the ICSI-derived diploid and haploid parthenotes.

**TABLE 4:** Chromosomal composition of bovine biparental diploid ICSI and haploid uniparental embryos.

Group	Embryos evaluated (cells)	No. of cells			
		1n	2n	Aneuploid	Total abnormal
ICSI	10 (28)	1 (4%)^a^	13 (46%)	14 (50%)	15 (54%)
Haploid partheno	20 (42)	15 (35%)^ab^	8 (19%)	19 (45%)	27 (64%)
Haploid androgeno	9 (11)	9 (81%)^bc^	0 (0%)	2 (19%)	2 (19%)

*ICSI, intracytoplasmic sperm injection using X-chromosome sorted sperm. Haploid partheno, haploid parthenogenetic embryo obtained by oocyte activation using ionomycin followed by cyclohexymide. Haploid androgeno, haploid androgenetic embryos generated using X-chromosome sorted sperm. 1n, haploid. 2n, diploid. Different superscripts denote significant differences within columns (p < 0.05).*

### Early Cleavage Events Are Not Affected in Haploid Androgenetic Embryos

**FIGURE 6:**
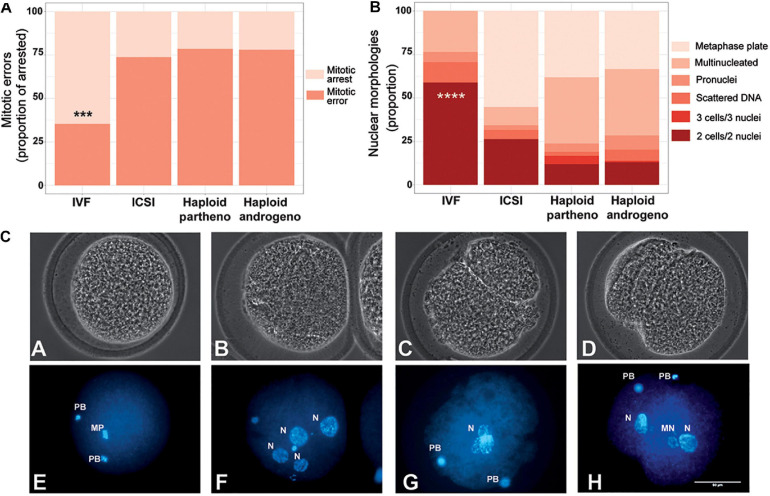
Developmental patterns of arrested embryos at 2- to 3-cell stage. **(A)** Proportion of embryos showing mitotic errors in relation to total number of arrested embryos. **(B)** Nuclear morphologies found in the zygotes showing developmental arrest. **(C)** Representative images of developmentally arrested embryos; **(A,E)** non-activated oocyte, **(B,F)** multinucleated zygote, **(C,G)** Anucleate blastomere, **(D,H)** Micronuclear formation. PB, polar body; mp, metaphase plate; n, nucleous; mn, micronucleus; ICSI, intracytoplasmic sperm injection using female-sorted semen; haploid partheno, haploid parthenogenetic embryos obtained by oocyte activation using ionomycin followed by cyclohexymide; haploid androgeno, haploid androgenetic embryo obtained by ICSI + oocyte enucleation. ^∗∗∗^*p* < 0.001, ^****^p < 0.0001. Scale bar 50 μm.

In humans, developmental anomalies during the first mitotic divisions of *in vitro*-derived embryos have been associated with poor gamete quality and *in vitro* handling (Hardy et al., 1993; Pelinck et al., 1998; Alikani et al., 2000; Babariya et al., 2017). In order to better define the aberrations associated with the poor development of haploid hAE, we first evaluated nuclear morphology of embryos that arrested between 1- to 3-cell stage after 48 h of culture. Apart from IVF-derived controls, all groups that underwent micromanipulation, such as ICSI, enucleation and chemical oocyte activation, had higher rate of mitotic anomalies (*p* < 0.001; Figure 6), suggesting that extensive *in vitro* manipulation of the oocyte is associated with early-stage developmental aberrations. Particularly, haploid androgenotes did not present additional anomalies when compared to ICSI-derived embryos, indicating that removal of the oocyte spindle at telophase does not further interfere with early cleavage. Together, these results indicate that mitotic errors during first cleavage divisions (i.e., 2nd and 3rd) were caused mostly by the micromanipulation procedures, confirming that the block in hAE progress to the blastocyst stage arises principally after reaching the 8-cell stage, and indicating that the arrest in development may be caused by abnormal gene expression following transcriptional activation.

### Haploid Androgenetic Embryos Show Dysregulated Gene Expression of X-Linked Genes and the KCNQ1 Locus

**FIGURE 7:**
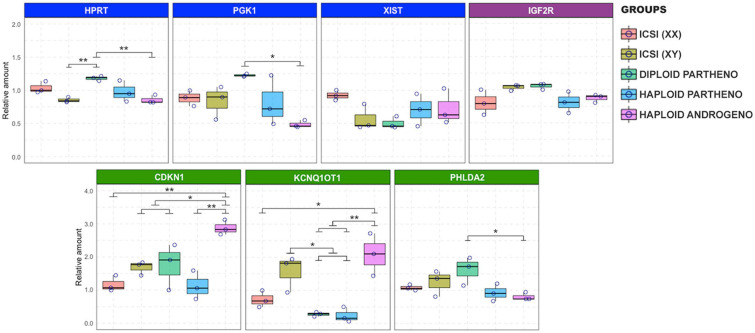
Relative transcript levels of different imprinted and X chromosome-linked genes on haploid and diploid 8-cell stage embryos obtained by ICSI, parthenogenetic activation or ICSI + enucleation. Blue boxes: X chromosome-linked genes: XIST, PGK1, and HPRT; green boxes: genes on the KNCQ1 locus: KCNQ1OT1, CDKN1, and PHLDA2. GAPDH, ACTB, and SF3A were used as housekeeping genes for normalizations. ^∗^*p* < 0.05, ^∗∗^*p* < 0.01.

Since haploid embryos possess exclusively maternal or paternal-derived chromosomes, genomic imprinting (autosomal or sex-related imprinting) offers another possible explanation for their poor early development (Latham et al., 2002). For instance, imprinting of the paternal X chromosome could potentially lead to development anomalies in haploid X chromosome-bearing androgenotes, as could the dysregulation of key imprinted genes that are either silenced or overexpressed due to parental-specific genomic imprints in hAE. We therefore analyzed the expression of X-linked genes and some imprinted genes that were previously described to undergo genomic imprinting in the bovine species (Jiang et al., 2015). This was achieved by using two different developmental stages, i.e., at 8- to16-cell (72 h or Day-3) and morula stage embryos (144 h or Day-6), to evaluate the expression levels at the time of the zygote genome activation (ZGA) and the moment of initial differentiation (i.e., compaction), respectively, when most haploid androgenotes arrested development (see Figure 2 and Supplementary File 2 where development to the blastocyst stage was severely limited in hAE). In addition, to analyze the effects of sex and ploidy on gene expression levels, we included different control groups, such as ICSI females (ICSI using X chromosome-sorted sperm), ICSI males (ICSI using Y chromosome-sorted sperm), and parthenotes (both diploid and haploid). At the time of ZGA (72 hpi), results indicate that the expression patterns of the X-linked genes XIST, PGK1, and HPRT were similar between haploid androgenotes, haploid parthenotes and biparental male and female embryos (Figure 7). In contrast, while the transcripts of the IGF2R imprinted gene did not show variation among groups, the imprinted genes of the KCNQ1 locus also showed significant differences in expression. KCNQ1OT1, the paternally expressed long non-coding RNA involved in regulating the KCNQ1 locus, was significantly upregulated in haploid androgenotes compared to parthenotes (haploids and diploids) and biparental female embryos (Figure 7). In contrast, parthenogenetic embryos barely expressed KCNQ1OT1, which confirms its imprinted nature of monoallelic paternal expression. Surprisingly, CDKN1C and PHLDA2, genes that are silenced on the paternal allele, were either upregulated or showed similar expression patterns in haploid androgenotes when compared to haploid parthenotes, respectively, indicating a dysregulation of the KCNQ1 locus at early stage after zygotic genome activation.

**FIGURE 8:**
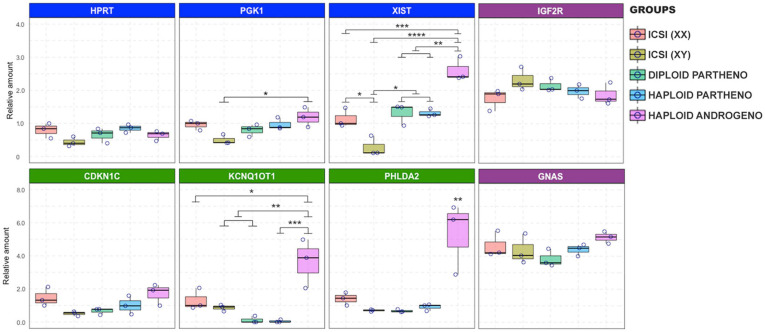
Relative transcript levels of different imprinted and X chromosome-linked genes on haploid and diploid morula stage embryos obtained by ICSI, parthenogenetic activation or ICSI + enucleation. Blue boxes: X-linked genes XIST, PGK1, and HPRT on X chromosome; green boxes: genes on the KNCQ1 locus KCNQ1OT1, CDKN1 and PHLDA2. GAPDH, ACTB, and SF3A were used as housekeeping genes for normalization. ^∗^*p* < 0.05, ^∗∗^*p* < 0.01, ^∗∗∗^*p* < 0.001, ^****^p < 0.0001.

At the Day-6 morula stage, effects on the expression of genes linked to the X chromosome and the KCNQ1 imprinted locus were exacerbated even beyond that observed on Day-3. XIST transcript levels were significantly overexpressed in the haploid androgenotes compared to all the control groups (Figure 8). Indeed, a 2.4-fold increase in XIST transcripts was observed in hAE compared to the ICSI-derived female embryos, the only other group containing a paternal X chromosome, indicating that androgenetic haploidy leads to a dysregulation of XIST transcription. Moreover, similar levels of XIST transcripts were observed between haploid parthenotes and diploid female embryos, which could be explained by the “self-diploidization” event already described in human haploid parthenogenetic embryos (Leng et al., 2017), and evidenced here by the low levels of cells that maintained a stable haploid karyotype in the parthenogenetic group (see Figure 5); or that parthenogenesis leads to the overexpression of XIST from the maternal X chromosome. However, HPRT and PGK1 transcript levels in haploid androgenotes did not differ from female ICSI embryos, indicating that other genes on the paternal X chromosome are unaffected by haploidy. As for the KCNQ1 imprinted locus, haploid androgenotes showed a significant upregulation of KCNQ1OT1 compared to all groups, including the ICSI-derived embryos that also contained a paternal allele. Surprisingly, PHLDA2, a paternally silenced gene was significantly overexpressed whereas CDK1NC levels were unaffected in haploid androgenotes (Figure 8). On the other hand, expression patterns of other imprinted genes, such as IGF2R and GNAS, were not altered in hAE, indicating that not all imprinted loci are disturbed on these embryos. The relative measure of the concentration of each target genes in the PCR reaction (*C*t values) can be found in Supplementary File 3 for X-linked and imprinted genes, and in Supplementary File 4 for DNMT1, DNMT3B, and TET1, and their corresponding reference genes. Altogether, these results show that the hAE have altered gene expression of some X chromosome and imprinted genes, suggesting that the developmental anomalies observed in haploid androgenotes at early stage of embryogenesis, i.e., during and soon after ZGA, are regulated epigenetically.

### DNMT3B Expression Is Downregulated in Haploid Androgenetic Embryos

**FIGURE 9:**
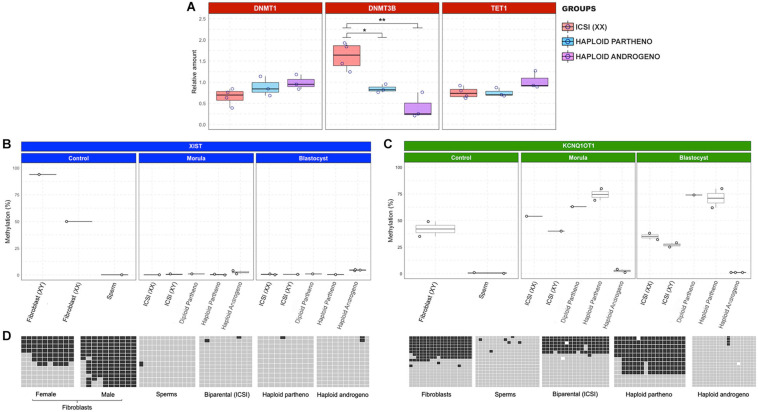
Relative transcript levels of DNA methylation-related enzymes and DNA methylation profiles of XIST and KCNQ1 DMR. **(A)** Relative transcript levels of DNMT1, DNMT3B, and TET1 in biparental diploid female (ICSI), haploid parthenogenetic and androgenetic embryos. **(B)** DNA methylation profiles of XIST and **(C)** KCNQ1 DMR in morula (pool of two embryos) and blastocyst (individual embryo) stages. Each point represents one biological replicates. **(D)** Representative image depicting the gene specific DNA methylation patterns of XIST and KCNQ1 DMR in somatic cells (fibroblast), sperm, and embryos. Fibroblast (XX), female fibroblast cells; Fibroblast (XY), male fibroblast cells; ICSI (XX), intracytoplasmic sperm injection using X chromosome sorted-sperm; ICSI (XY), intracytoplasmic sperm injection using male sorted-sperm; haploid partheno: haploid parthenogenetic embryo obtained by oocyte activation using ionomycin followed by cyclohexymide; haploid androgeno, haploid androgenetic embryo obtained by ICSI + oocyte enucleation. ^∗^*p* < 0.05, ^∗∗^*p* < 0.01.

Previous studies have shown that bovine *in vitro* culture conditions at the early embryo stages induce changes to imprinted gene expression through abnormal expression of DNA methyltransferases (Wei et al., 2011). Owing to the dysregulated transcript expression observed in hAE, specifically in genes from the KCNQ1 locus and X chromosome, we analyzed the expression of enzymes related to the maintenance (DNMT1) and *de novo* (DNMT3B) DNA methylation, as well as DNA demethylation (TET1) in haploid (parthenogenetic and androgenetic) and biparental (ICSI) Day-6 morula stage embryos. Although no differences were observed between the levels of DNMT1 and TET1 transcripts, DNMT3B expression was significantly downregulated in androgenetic and parthenogenetic haploid groups compared the biparental control embryos (Figure 9A). This indicates that developmental events that require *de novo* methylation may be impaired in haploid embryos. In contrast, unaltered expression levels of DNMT1 and TET1 demonstrate that DNA methylation maintenance and active demethylation are not affected in both androgenetic and parthenogenetic haploid groups.

### Methylation Patterns of the XIST, KCNQ1OT1, and H19 DMRs Are Unaltered in Haploid Androgenetic Embryos

To evaluate whether the abnormal expression of genes from the X chromosome and KCNQ1 locus was associated with alterations in methylation patterns of differentially methylated regions (DMR), we performed bisulfite sequencing of the XIST and KCNQ1 DMR in morula and blastocyst stage embryos. First, in order to evaluate whether hAE embryos maintain or not methylation inherited from sperm, we performed the analysis of the paternally methylated H19 DMR in sperm, fibroblasts, biparental, haploid parthenogenetic and androgenetic embryos. We found hypomethylation of haploid parthenotes (0%) and approximately 50% methylation in biparental (ICSI) embryos and fibroblasts (65 and 41%, respectively), and 100% in sperm (Supplementary Figure 3). As expected, we found hypermethylation of the H19 DMR in hAE (84–100%), reflecting their androgenetic origin (Supplementary Figure 3).

As observed in other mammalian species, the bovine XIST DMR region was 50% methylated in adult female fibroblasts and hypermethylated in adult male fibroblasts, supporting the notion of dosage-compensation by X chromosome inactivation in female somatic tissues (Figures 9B,D). In contrast, the XIST DMR was hypomethylated in sperm and biparental embryos. Besides, XIST DMRs in haploid androgenetic and parthenogenetic (haploid and diploid) embryos were all hypomethylated. These results indicate that the upregulation of XIST expression in hAE cannot be explained by alterations to the methylation status of its DMR.

As expected for DMRs controlling imprinted loci, the KCNQ1 DMR was hypomethylated in the male gamete, approximately 50% methylated in fibroblast and biparental (ICSI-derived) embryos (Figures 9C,D). Moreover, diploid and haploid parthenogenetic embryos at the morula and blastocysts stage showed increased methylation of the KCNQ1 DMR, indicating a hypermethylation of the maternal allele. In contrast, androgenetic embryos showed a hypomethylated pattern at morula and blastocyst stages that resembled the patterns observed in spermatozoa typical of the KCNQ1 imprinted locus.

Together, since the KCNQ1 DMR in hAE maintains the hypomethylated patterns found in sperm, these results indicate that androgenetic haploidy leads to the dysregulation of the paternal-specific long non-coding transcript and other imprinted genes from the KCNQ1 locus by epigenetic mechanisms other than the methylation status of its DMR.

## Discussion

Here, we analyzed different methods for production of bovine hAE and the potential causes of their limited ability to develop to the blastocyst stage. Our results indicate anomalies in expression of genes from the X chromosome and KCNQ1 imprinted loci, suggesting the involvement of epigenetic regulators in the developmental constraints to haploid androgenotes in the bovine species.

Initially, we evaluated a range of methods for the removal of the oocyte spindle (enucleation), i.e., before and after *in vitro* fertilization, on subsequent development of haploid androgenetic early stage embryos (Latham et al., 2002; Lagutina et al., 2004; Vichera et al., 2011). Haploid androgenetic embryos produced by both these methods showed similar cleavage rates, but development to the blastocyst stage was severely affected. In agreement with our findings, previous studies have reported that most the bovine hAE arrest after the first cell divisions and that only a few of them reach the blastocyst stage (Lagutina et al., 2004; Vichera et al., 2011). In mice, hAE produced by fertilization of enucleated oocytes also showed limited blastocyst development (11%) compared to the IVF group (90%) (Kono et al., 1993). Nonetheless, our results showed that hAE development is enhanced by enucleation post-IVF. In agreement with these findings, others have indicated that enucleation during the telophase stage of second meiosis (TII) shows advantages over enucleation at the metaphase II stage (Bordignon and Smith, 1998; Lee and Campbell, 2006; Kuznyetsov et al., 2007; Sagi et al., 2019).

However, although the production of hAE is feasible by using conventional IVF, we found a high proportion of multinucleated zygotes, indicative of polyspermic fertilization, as has been previously reported in mice (Kono et al., 1993) and cattle (Lagutina et al., 2004). Polyspermic fertilization after bovine IVF is known to occur in between 5 and 25% of the presumptive zygotes (Roh et al., 2002; Coy et al., 2005) making it an unreliable approach for producing consistently normal samples of hAE. Therefore, we decided to switch to using ICSI instead of IVF to avoid potential issues with polyspermic fertilization. In agreement with previous reports (Latham et al., 2002; Lagutina et al., 2004; Vichera et al., 2011; Yang et al., 2012), our results showed that haploid androgenetic zygotes can be reliably and effectively produced by combining ICSI followed by removal of the oocyte’s TII spindle.

Cleavage rate and cell number of hAE after 48 h of culture indicated that micromanipulation (ICSI and TII enucleation) does not influence the initial mitotic divisions of early embryonic development. Nonetheless, although androgenetic haploidy does not have an impact on development up to around the third or fourth mitotic division, many hAE underwent developmental arrest at the 8-cell stage, i.e., at the time of zygotic genome activation (ZGA). Indeed, the absence of the maternal genome in androgenetic embryos raises the possibility of a general defect in ZGA that could impact on the development shortly after the 8-cell stage, as we have observed in this study. Recently it has been reported that uniparental porcine embryos show an unusual chromosomic decompartmentalization and a non-synchronized nuclear reprogramming, implying a severe dysregulation of ZGA (Li et al., 2020). Similarly, by using single-cell RNA-seq, another study showed that uniparental human embryos exhibit variable and fewer patterns of EGA associated with a reduced depletion of maternal transcripts (Leng et al., 2019). Thus, exploring the possibility of a general defect in ZGA process that could impact on bovine hAEs development shortly after the 8-cell stage not only will provide a valuable knowledge in uniparental reproduction studies, but also address the relationship between ZGA regulators and uniparental development in the bovine species. In addition, androgenetic embryos that progressed beyond ZGA underwent a second developmental arrest at the morula stage. Similar results have been reported in cattle (Winger et al., 1997; Vichera et al., 2011), sheep (Matsukawa et al., 2007), mice (Kono et al., 1993; Latham et al., 2002; Hu et al., 2015a,b), and humans (Kuznyetsov et al., 2007; Sagi et al., 2019). Further development to the blastocyst stage, of both haploid parthenotes and androgenotes was severely limited, evidencing a deleterious effect of haploidy on early stages of embryogenesis.

On the other hand, when we produced hAE using Y chromosome-sorted sperm, the embryonic development was arrested at cleavage stages before compaction, confirming previous observations in mice (Latham et al., 2002; Yang et al., 2012). Similarly, we showed that bovine haploid androgenotes derived from X chromosome-sorted sperm develop poorly, and only rarely reach the blastocyst stage. Moreover, since sex sorting techniques are typically 90% accurate (Sharpe and Evans, 2009), these results indicate that the poor development of haploid androgenotes produced using X chromosome-sorted sperm cannot be explained by erroneous use of sperm carrying the Y chromosome. Moreover, assessment of total cell number and blastocyst morphology in haploid androgenotes, both positively correlated with blastocyst quality (Sagirkaya et al., 2006; Kong et al., 2016), showed fewer cells and delayed blastulation, compared not only to biparental controls but also to the haploid parthenotes, suggesting that the haploid paternal genome is less capable to support early development than the haploid maternal genome.

The analysis of chromosomal constitution in morula-stage embryos showed that aneuploidy levels were higher in haploid parthenotes than in androgenotes, indicating that chromosomal segregation errors are not likely the cause for the limited early development of hAE. Since bovine centrosomes are inherited from the sperm at fertilization and are responsible for organizing the mitotic spindle of the zygote (Long et al., 1993; Navara et al., 1994, 1995, 1996; Sutovsky et al., 1996a,b), it is likely that the sperm centrosome introduced during ICSI in hAE is capable of forming a stable mitotic spindle, regardless of the removal of the oocyte TII spindle, which may explain their ability to maintaining a stable karyotype during the subsequent mitotic divisions up to the morula stage. It will be very important in the future to further elucidate if bovine hAE maintain a stable karyotype at the blastocyst stage, especially if a forthcoming objective is to derive androgenetic hESCs. Nonetheless, aneuploidy levels in ICSI-derived embryos were also quite high, suggesting that the oocyte’s spindle itself and not the absence of the spermatozoa’s centrosome is responsible for the elevated levels of aneuploidy observed in the haploid parthenotes. It is likely that slaughterhouse-derived bovine oocytes obtained from either aged females and/or by suboptimal *in vitro* maturation systems contain unstable meiotic spindles that lead to chromosomal segregation errors during early embryogenesis (Li M. et al., 2014; Shomper et al., 2014).

According to Matsukawa et al. (2007), hAE that undergo a developmental arrest before first cleavage commonly present micronuclei and pycnotic nuclear formation. For this reason, we decided to evaluate the nuclear morphology of arrested zygotes. Our results indicated that micromanipulation procedures are related to the early developmental defects. In agreement with these results, numerous studies have associated bovine ICSI with several developmental aberrations such as insufficient sperm head decondensation (Rho et al., 1998; Malcuit et al., 2006), delayed pronuclear formation (Aguila et al., 2017), and altered gene expression (Arias et al., 2015). However, although harmful effects observed after ICSI could contribute to the early developmental failure of androgenotes, they do not explain the development arrest observed beyond ZGA. Accordingly, a study by Latham et al. (2002) indicated that the sperm injection procedure used for ICSI does not influence *in vitro* development. Altogether, such results support the notion that the reduced developmental potential to the blastocyst stage of bovine haploid androgenotes is not caused by the handling artifacts during micromanipulation but are rather related to impairments resulting from gene expression anomalies after ZGA.

Silencing of one of the X chromosomes in biparental female embryos is regulated by the expression of XIST, a non-coding RNA that acts as a major effector on X chromosome inactivation (XCI), and the methylation of XIST prevents its expression. In the mouse, following zygotic genome activation (ZGA), only the paternal X chromosome (Xp) undergoes Xist-mediated silencing (Xip), which is maintained in the trophectoderm at the blastocyst stage, but in epiblast cells the inactive Xp is reactivated and later, upon implantation, epiblast cells undergo random XCI (Rebuzzini et al., 2020). However, the pattern of XCI in other mammalian species is not clear yet. For example, in preimplantation human and bovine embryos, XCI does not follow an imprinted inactivation as in the mouse, and the precise mechanism of dosage compensation continues being debated (Bermejo-Alvarez et al., 2010; Saiba et al., 2018). Nonetheless, the effects of paternal haploidy on expression during early embryogenesis of X chromosome and imprinted genes has not yet been evaluated in bovine species. Moreover, uniparental haploid embryos possess only one copy of either the paternal or maternal genomes and, therefore, lack imprinted gene transcripts expressed monoallelically from either one or the other. For instance, the KCNQ1 imprinted domain is one of the largest known imprinted clusters, and its altered imprinting has been associated with a fetal overgrowth pattern known as large offspring syndrome or LOS (Lee et al., 1999; Chen et al., 2015). The KCNQ1 locus is regulated by a differentially methylated region, i.e., KvDMR1, located in the promoter of the non-coding KCNQ1OT1 gene and methylated exclusively on the maternal allele. Therefore, KCNQ1OT1 is paternally expressed and negatively regulates the expression of several maternally expressed genes of the KCNQ1 locus, including CDKN1C, KCNQ1, and PHLDA2 (Ager et al., 2008). Thus, differential expression of X-linked and imprinted genes, can help to address the causes behind the limited *in vitro* developmental potential of haploid androgenotes.

Although our data revealed similar expression levels of the XIST gene at the time of ZGA, hAE showed downregulation of HPRT and PGK1 transcription compared to diploid parthenotes, indicating that the expression of some X chromosome genes can be altered as soon as the 8-cell stage. In contrast, XIST was substantially overexpressed in the hAE at the morula stage while the levels of genes subject to XCI suggest lacking dosage compensation for PGK1, while HPRT dosage appears unaffected in hAE at this stage. While the presence of XIST transcripts has been reported as early as the 2-cell stage (Mendonca et al., 2019), microarray and RNA-seq analyses of bovine blastocysts demonstrated higher expression of X-linked genes in female compared with male and parthenogenetic embryos, indicating that dosage compensation is initiated later than in other mammals (Bermejo-Alvarez et al., 2010; Min et al., 2017). Although a recent report has indicated that XIST accumulation and XCI in bovine embryos begins at the morula stage, XIST colocalization with repressive marks, such as H3 lysine 27 trimethylation, on histones was only detected in Day-7 blastocysts, indicating that complete XCI is only partially achieved at the blastocyst stage (Yu et al., 2020). Although XIST accumulation did not lead to globally reduced expression of X-linked genes and XCI is only partially achieved at the blastocyst stage (Bermejo-Alvarez et al., 2010; Yu et al., 2020), the relevance of the dysregulated expression of this long-non-coding RNA in hAE needs further clarification of its impacts on chromosome-wide downregulation of gene expression and on the embryonic demise. Latham et al. (2002) reported elevated expression of Xist RNA in haploid mouse androgenotes, and a similar pattern for the PGK1 gene, suggesting either that haploid androgenotes undergo deficient XCI, or that the embryos that initiate the XCI process begin to die soon thereafter. Haploid androgenotes with the greatest degree of Xist RNA expression, PGK1 gene repression, and repression of other X-linked genes may die within a narrow period of time just after ZGA, which is in line with our findings that the majority of haploid androgenotes fail to progress to the morula and blastocyst stages.

To our knowledge, this is the first study to assess the expression of genes from the KCNQ1 imprinted domain in haploid androgenetic mammalian embryos. In mice, KCNQ1OT1 is paternally expressed as early as two-cell stage and maintained throughout preimplantation development, while the ubiquitously imprinted genes KCNQ1 and CDKN1C are paternally repressed at the morula/blastocyst stages. By contrast, placentally imprinted genes TSSC4 and CD81 from the same domain show biallelic expression at the blastocyst stage (Umlauf et al., 2004; Lewis et al., 2006). In this study, KCNQ1OT1 and PHLDA1 were overexpressed in haploid androgenetic morula stage embryos. Also, CDKN1C expression was unexpectedly upregulated in androgenotes compared to male diploid embryos. As discussed earlier, haploid androgenotes that were able to progress up to morula stage might have escaped from KCNQ1OT1 silencing or those with the greatest degree of imprinting repression arrested just after ZGA. Thus, the abnormal expression of the KCNQ1 imprinted domain could potentially affect the development and differentiation of haploid androgenetic early-stage embryos. Finally, IGF2R and GNAS, two paternal imprinted genes (Jiang et al., 2015), showed similar expression levels among androgenotes and control embryos, suggesting either that their imprinting was relaxed or, as previously reported, that the monoallelic expression in ruminant species may not be required for most imprinted genes during early embryonic development (Cruz et al., 2008).

DNA cytosine methylation is one of the most important modifications in the epigenetic genome and plays essential roles in various cellular processes, including genomic imprinting, XCI, retrotransposon silencing, as well as regulation of gene expression and embryogenesis (Reik et al., 2001). The addition of methyl groups to cytosine residues in preimplantation bovine embryos is catalyzed by DNA methyltransferases for maintenance (e.g., DNMT1) and DNMT3A and DNMT3B for *de novo* methylation (Smith et al., 2012; Ross and Sampaio, 2018). Active DNA demethylation at early development in mammals has been ascribed to TET activity (Iqbal et al., 2011). In cattle, the presence of DNMT3B has been reported as a key element for the control of methylation levels at advanced preimplantation stages, specifically at morula and blastocyst stages (Ross and Sampaio, 2018). Besides, among the factors required for demethylation process, TET1 is the predominant expressed enzyme after zygote genome activation (Bakhtari and Ross, 2014). Our results indicate that haploid and biparental embryos show similar levels of DNMT1 and TET1 transcripts. The zygotically expressed form of DNMT1 maintains the methylation of imprints at each cell cycle during early embryonic development in mammals (Hirasawa et al., 2008; Kurihara et al., 2008), suggesting that hAE are able to maintain their methylation imprints. Conversely, DNMT3B was deficient in both haploid (androgenetic and parthenogenetic) groups. In accordance with our results, a previous study in mice demonstrated that DNMT3B was significantly downregulated in haploid androgenetic ESCs lines, affecting its production and derivation (He et al., 2018), suggesting that proper DNMT3B activity and the content of methylation is essential for the development of mammalian hAE. In cattle, DNMT3A, DNMT3B, and DNMT3L are expressed in oocytes during the critical period of DNA methylation imprint acquisition (O’Doherty et al., 2012). Also, DNMT transcripts (DNMT1, DNMT2, DNMT3A, and DNMT3B transcripts) can be found at the 2-cell, 4-cell, 8-cell, 16-cell, morula, and blastocyst stage in bovine preimplantation embryos (Golding and Westhusin, 2003). As in mice, dynamic changes in DNA methylation reprogramming are associated with the reestablishment of genomic imprinting and acquisition of totipotency in bovine early zygotes and embryos (Dean et al., 2001; Park et al., 2007; Dobbs et al., 2013), and can be easily altered by environmental conditions such as *in vitro* manipulation technologies (Hou et al., 2007; Heinzmann et al., 2011). Reports have indicated that the period of demethylation from 2- to the 6–8 cell stage is followed by a period of *de novo* methylation from the 8–16 cell stages up to the blastocyst (Dean et al., 2001; Dobbs et al., 2013) and that this dynamic methylome is closely related to dynamic changes to the expression of methyltransferases (Dobbs et al., 2013; Duan J.E. et al., 2019; Ivanova et al., 2020). In synchrony with the onset of *de novo* methylation of bovine blastocysts (Dean et al., 2001; Dobbs et al., 2013; Jiang et al., 2014), DNMT3A and DNMT3B transcripts augment before the time of ZGA (Duan J. et al., 2019), and also from the morula to the blastocyst stage (Ivanova et al., 2020), suggesting a role during early and later stages of bovine preimplantation development. In mice, DNMT3B deficiency severely damages post-implantation development (Okano et al., 1999). Also, inactivation of DNMT3B in biparental embryos induces global DNA hypomethylation (Kato et al., 2007; Auclair et al., 2014; Fu et al., 2020). Although it remains unknown whether lower DNMT3B expression promotes global hypomethylation in bovine hAE.

Finally, we performed a gene specific bisulfite sequencing in order to analyze whether DMR abnormal methylation patterns could be related to the overexpression of XIST and KCNQ1OT1 genes in hAE. As a control for a paternally methylated DMR we used H19 gene. As expected, the H19 DMR was hypermethylated in haploid androgenetic embryos, suggesting that bovine hAE retain imprinted patterns inherited from the sperm, as seen in other animal models (Yang et al., 2012, 2013; Li W. et al., 2014). As for the XIST DMR, all groups were equally demethylated at both the morula and blastocyst stages, indicating that DMR methylation occurs at later stages of embryogenesis. Indeed, in contrast to somatic cells, loss of methylation of the XIST DMR in embryonic cells is not related to transcriptional activity (Beard et al., 1995). Surprisingly, although both contain a single maternal X chromosome, haploid parthenotes contained 6 times more XIST transcripts compared to male ICSI-derived embryos. Moreover, the twofold overexpression of XIST in haploid androgenotes compared to the parthenotes, regardless of their hypomethylated DMR, further supports the notion that epigenetic mechanisms other than DMR methylation control transcription from this locus in early embryos. It is likely that the XIST DMR undergoes methylation later during bovine embryonic development, since the onset of XCI initiates at the blastocyst stage (Yu et al., 2020). As for the KCNQ1 DMR, biparental somatic cells and embryos showed close to 50% methylation while embryos carrying only maternal alleles (haploid and diploid parthenogenetic embryos) were hypermethylated. Moreover, embryos from the haploid androgenetic group were hypomethylated, resembling the imprinted profile inherited from the sperm (Robbins et al., 2012), suggesting that the overexpression of the maternally (PHLDA2) and paternally expressed (KNCQ1OT1) genes were not related to their imprinted status. In a similar fashion, others have reported that parental imprints are generally maintained in haploid androgenetic ESC, but the expression of some imprinted genes has a distorted correlation with their parent-of-origin methylation status (Yang et al., 2012; Li W. et al., 2014; Sagi et al., 2019). In this line, Sotomaru et al. (2002) and Hu et al. (2015b) suggest that the paternal and maternal alleles are required for the appropriate expression of some imprinted genes. The lack of mechanisms acting in *trans* between both parental alleles could be behind the loss of parental-specific expression of some imprinted genes in uniparental mammalian embryos (Ogawa et al., 2006).

## Conclusion

This study has shown that bovine haploid androgenetic zygotes can be effectively produced by combining ICSI followed by mechanical enucleation of the oocyte spindle after the extrusion of the second polar body. Besides, micromanipulation effects and chromosomal abnormalities are not main factors affecting the development of bovine hAE. On the other hand, although the cause of the arrest remains unclear, and that neither X-linked, imprinted, nor some other gene expression defects can be ruled out at this time, we have shown that the inefficient development to the blastocyst stage of haploid androgenetic bovine embryos is associated with the abnormal expression of key factors involved in X chromosome activity and genomic imprinting. In order to obtain a better understanding of epigenetic regulation in the bovine haploid androgenetic model, future studies will be aimed at a more in-depth analysis of the global epigenetic features involving global transcriptomic and methylation analysis as well the study of repressive epigenetic marks in haploid embryos.

## Data Availability Statement

The raw data supporting the conclusions of this article will be made available by the authors, without undue reservation.

## Ethics Statement

The animal study was reviewed and approved by Comité d’éthique de l’utilisation des animaux, Université de Montréal.

## Author Contributions

LA, JT, and LS contributed to conception and design of the study. LA, JT, JS, MG, KM, and AH contributed with experimental procedures, including embryo production, manipulations, and molecular analysis. LA, JT, and LS wrote the manuscript. All authors contributed to manuscript revision, read, and approved the submitted version.

## Conflict of Interest

The authors declare that the research was conducted in the absence of any commercial or financial relationships that could be construed as a potential conflict of interest.

## Supplementary Material

Supplementary Figure 1 Proportion of cleaved embryos at the 8-cell stage at 48 h of culture. IVF, *in vitro* fertilized; ICSI, intracytoplasmic sperm injection using female-sorted semen; haploid partheno, haploid parthenogenetic embryos obtained by oocyte activation using ionomycin followed by cyclohexymide; haploid androgeno, haploid androgenetic embryo obtained by ICSI + oocyte enucleation.

Supplementary Figure 2 Morphological assessment of haploid androgenetic embryos produced with sperm carrying Y-chromosome at 144 h of culture. Representative **(A)** morphologies and **(B,C)** nuclear staining of embryos harvested at Day-6 (144 h) of culture.

Supplementary Figure 3 DNA methylation percentages of the H19 DMR in sperm, fibroblasts, biparental, haploid parthenogenetic and androgenetic embryos. Representative **(A)** sperm, **(B)** fibroblast, **(C)** biparental (ICSI), **(D)** haploid parthenogenetic and **(E–G)** androgenetic embryos. A pool of 2 morula-stage embryos were used for each analysis. ICSI, intracytoplasmic sperm injection. Haploid parthenogenetic: haploid embryo obtained by oocyte activation using ionomycin followed by cyclohexymide; haploid androgenenetic, haploid embryo obtained by ICSI + oocyte enucleation.
