# Supplementary figures and images for: Dysregulated Gene Expression of Imprinted and X-Linked Genes: A Link to Poor Development of Bovine Haploid Androgenetic Embryos
Source: Front Cell Dev Biol. 2021 Mar 18;9:640712. doi: 10.3389/fcell.2021.640712 (PMC8044962; doi:10.3389/fcell.2021.640712)

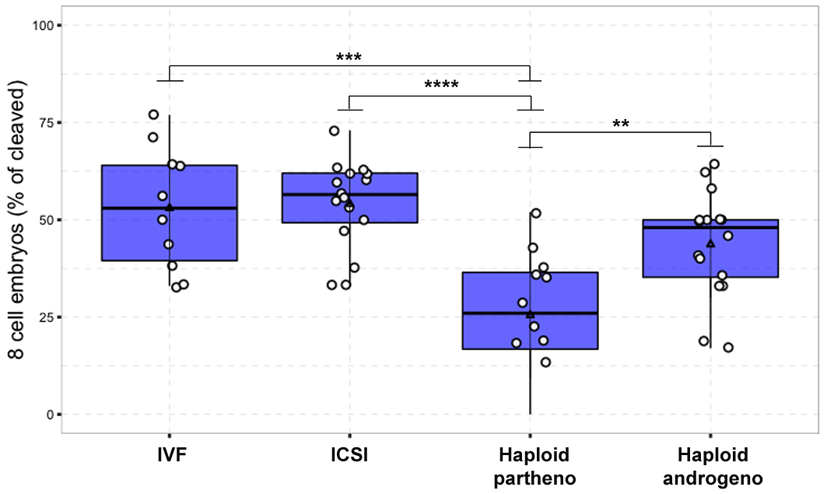

Supplement: Supplementary Figure 1 — Proportion of cleaved embryos at the 8-cell stage at 48 h of culture. IVF, in vitro fertilized; ICSI, intracytoplasmic sperm injection using female-sorted semen; haploid partheno, haploid parthenogenetic embryos obtained by oocyte activation using ionomycin followed by cyclohexymide; haploid androgeno, haploid androgenetic embryo obtained by ICSI + oocyte enucleation. [file Image_1.TIF]

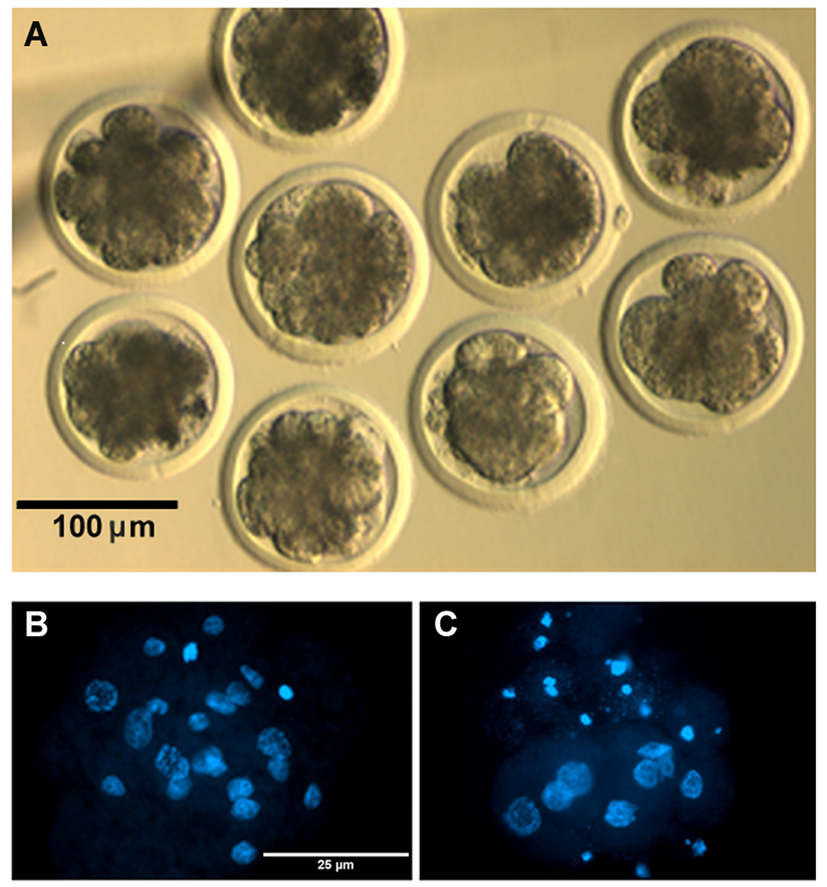

Supplement: Supplementary Figure 2 — Morphological assessment of haploid androgenetic embryos produced with sperm carrying Y-chromosome at 144 h of culture. Representative (A) morphologies and (B,C) nuclear staining of embryos harvested at Day-6 (144 h) of culture. [file Image_2.TIF]

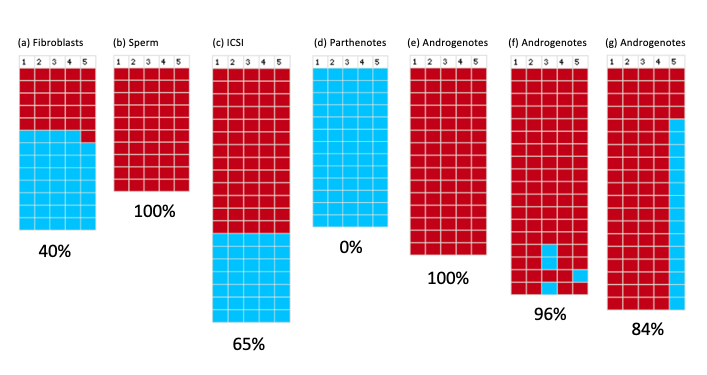

Supplement: Supplementary Figure 3 — DNA methylation percentages of the H19 DMR in sperm, fibroblasts, biparental, haploid parthenogenetic and androgenetic embryos. Representative (A) sperm, (B) fibroblast, (C) biparental (ICSI), (D) haploid parthenogenetic and (E–G) androgenetic embryos. A pool of 2 morula-stage embryos were used for each analysis. ICSI, intracytoplasmic sperm injection. Haploid parthenogenetic: haploid embryo obtained by oocyte activation using ionomycin followed by cyclohexymide; haploid androgenenetic, haploid embryo obtained by ICSI + oocyte enucleation. [file Image_3.TIFF]
